# Supplementary material for: Investigation of the Amide Linkages on Cooperative Supramolecular Polymerization of Organoplatinum(II) Complexes
Source: Molecules. 2021 May 10;26(9):2832. doi: 10.3390/molecules26092832 (PMC8126204; doi:10.3390/molecules26092832)
Supplement: Supplementary file 1 [file molecules-26-02832-s001.zip › molecules-1183220-supplementary.pdf]

Supporting Information

Investigation of the Amide Linkages on Cooperative Supramolecular Polymerization of Organoplatinum(II) Complexes

Mingliang Gui, Yifei Han, Hua Zhong, Rui Liao,* and Feng Wang*

CAS Key Laboratory of Soft Matter Chemistry, Department of Polymer Science and Engineering, University of Science and Technology of China, Hefei, Anhui 230026 P. R. China, E-mail: drfwang@ustc.edu.cn (F.W.); rliao@ustc.edu.cn (R.L.)

1. *Supramolecular polymerization behaviors of (S)-**2** in apolar media* S3
2. *Comparison of supramolecular polymerization thermodynamics between (S)-**1** and (S)-**2*** S8
3. *Majority-rules for supramolecular polymers derived from (S)-**2**/(R)-**2*** S9
4. *Supramolecular polymerization behaviors of (S)-**3*** S10
5. *Synthesis and structural characterization of (S)-**2** and (S)-**3*** S22

*1. Supramolecular polymerization behaviors of (S)-**2** in apolar media*

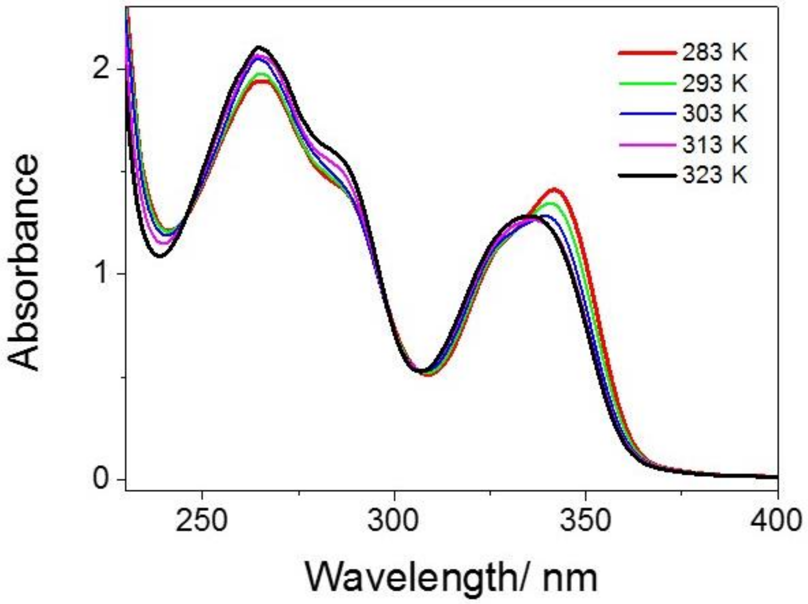

Figure S1. Variable-temperature UV–Vis spectra of (*S*)-**2** from 323 K to 283 K in MCH at the monomer concentration of 0.04 mM. It shows bathochromic shift for the IL transition band (λ_{max}: from 335 nm to 342 nm), upon lowering the temperature to 283 K. Moreover, two isosbestic points locating at 298 and 335 nm exist upon varying the temperature. The phenomena illustrate reversible transition between the monomeric and aggregated states of (*S*)-**2**.

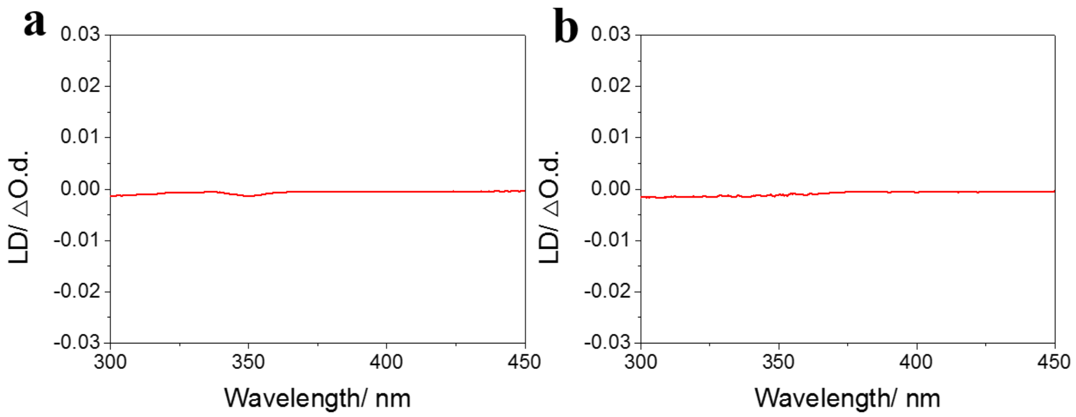

Figure S2. LD spectra of (a) (*S*)-**2** and (b) (*R*)-**2** (*c* = 0.04 mM, 283K). As can be seen, no LD signals are detected, demonstrating the measured CD signals (as shown in Fig.**1** in the main text) are real to reflect the supramolecular chirality.

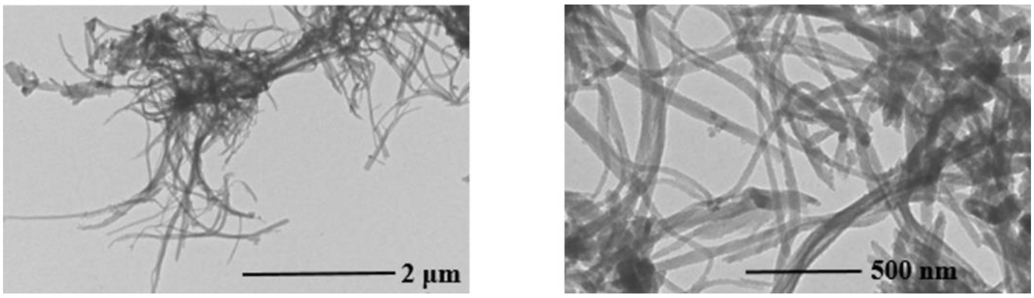

Figure S3. TEM images of (*S*)-**2** (copper grid, 0.06 mM). It shows the intertwined nanofibers with several microns in length and approximately 70 nm in width.

*2. Comparison of supramolecular polymerization thermodynamics between (S)-**1** and (S)-**2***

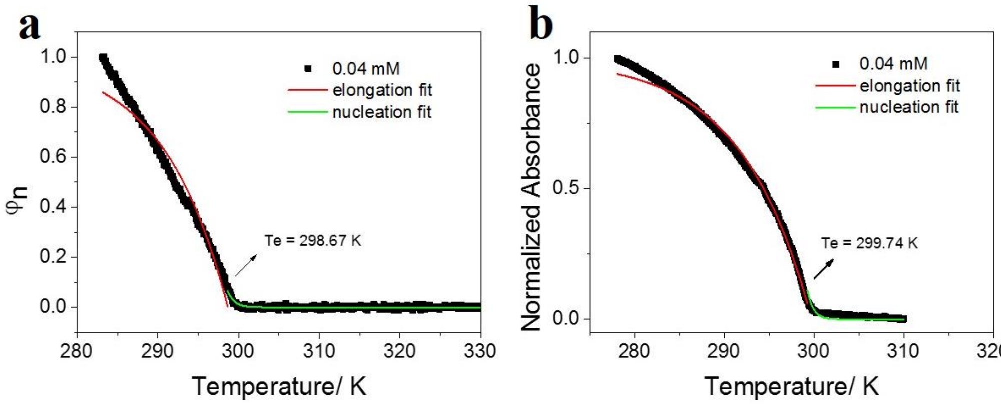

Figure S4. (a) Normalized CD intensity of (*S*)-**2** *versus* temperature at 350 nm in MCH (*c* = 0.04 mM). (b) Normalized UV–Vis intensity of (*S*)-**2** *versus* temperature at 348 nm in MCH (*c* = 0.04 mM). Depending on the UV–Vis melting curve, T_e (critical elongation temperature) value is determined to be 299.7 K, while the h_e (enthalpy release upon elongation) value is –96.5 kJ mol^{-1}. Similar values were obtained for the CD melting curve under the same circumstances (T_e: 298.7 K, h_e: –93.5 kJ mol^{-1}).

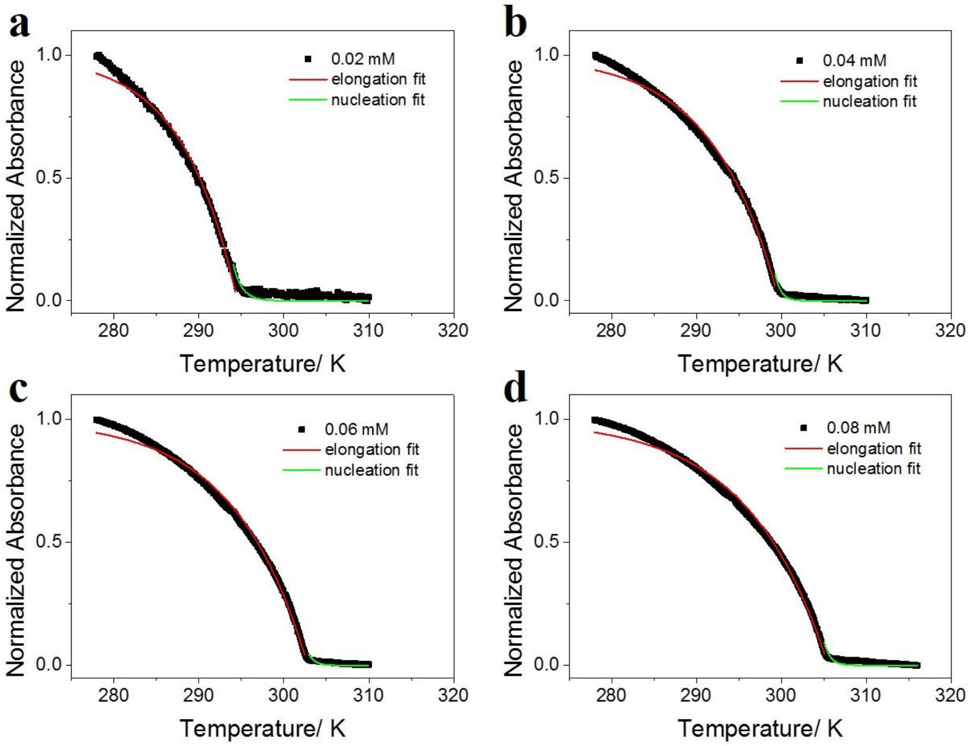

Figure S5. Normalized UV–Vis absorbance intensity of (*S*)-**2** in MCH at different monomer concentration: (a) 0.02 mM; (b) 0.04 mM; (c) 0.06 mM; (d) 0.08 mM. The red and green lines denote mathematical fitting of the curves by Meijer–Schenning–van der Schoot model. The quantitative thermodynamic values are shown in **Table S1**.

Table S1. **Thermodynamic parameters of (*S*)-2 obtained by fitting temperature-dependent UV–Vis data.**

(*S*)-**2**	0.02 mM	0.04 mM	0.06 mM	0.08 mM
T_e (K)	294.5	299.7	302.8	305.5
h_e (kJ mol^{-1})	–113	–96.5	–89.0	–82.9

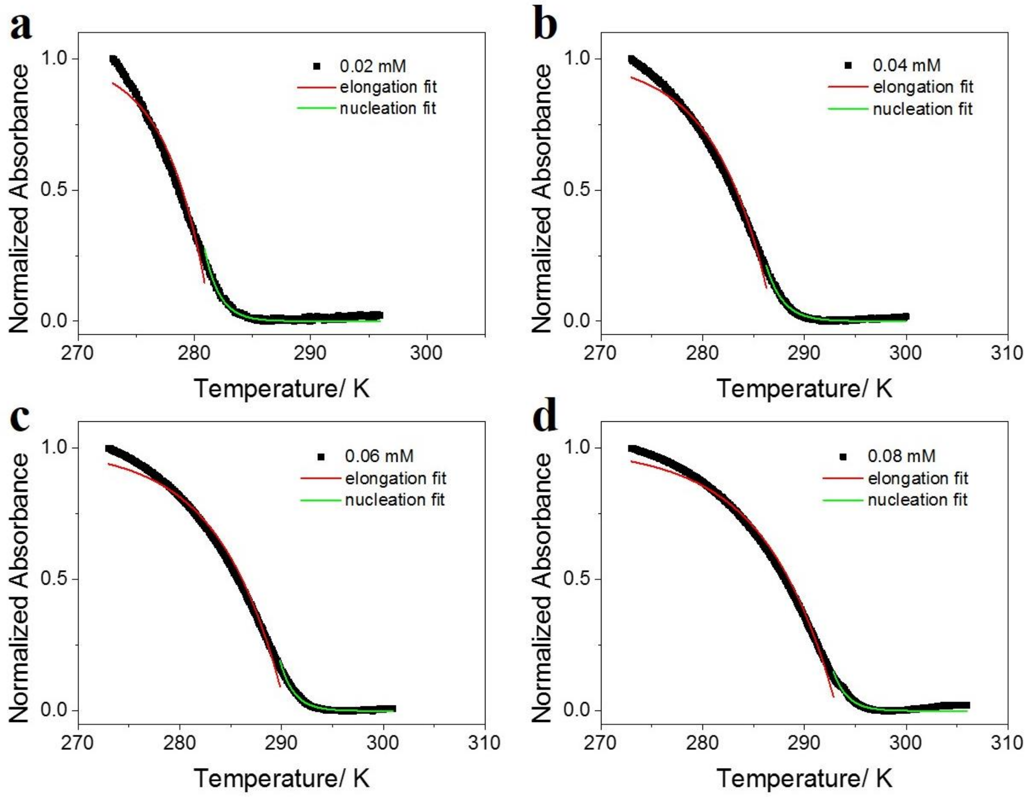

Figure S6. Normalized UV–Vis absorbance intensity of (*S*)-**1** in MCH at different monomer concentration: (a) 0.02 mM; (b) 0.04 mM; (c) 0.06 mM; (d) 0.08 mM. The red and green lines denote mathematical fitting of the curves by Meijer–Schenning–van der Schoot model. The quantitative thermodynamic values are shown in **Table S2**.

Table S2. **Thermodynamic parameters of (*S*)-1 obtained by fitting temperature-dependent UV–Vis data.**

(*S*)-**1**	0.02 mM	0.04 mM	0.06 mM	0.08 mM
T_e (K)	281.4	287.0	290.5	293.2
h_e (kJ mol^{-1})	–184	–129	–113	–105

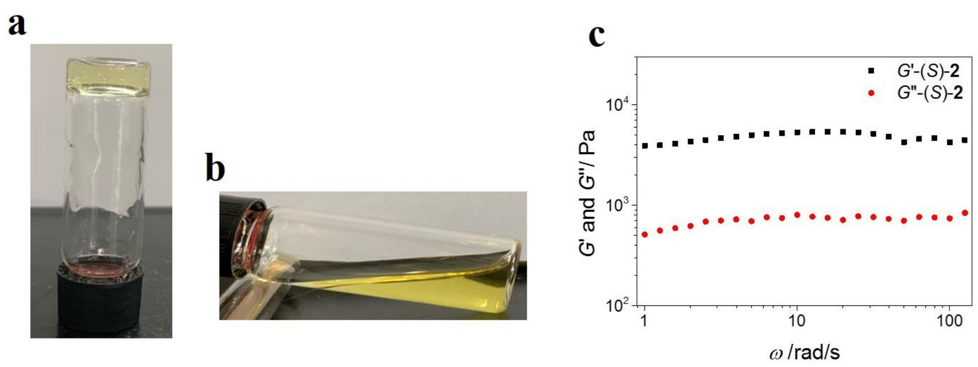

Figure S7. (a) Organogel of (*S*)-**2** at the concentration of 11.0 mM in MCH. (b) Failure for gel formation in terms of (*S*)-**1** at the concentration of 20 mM in MCH. (c) Elastic modulus *G*' (black symbols) and viscous modulus *G*'' (red symbols) for the gels derived from (*S*)-**2** (20.0 mM in MCH) as a function of oscillation frequency at 298K with a strain of 0.2%.

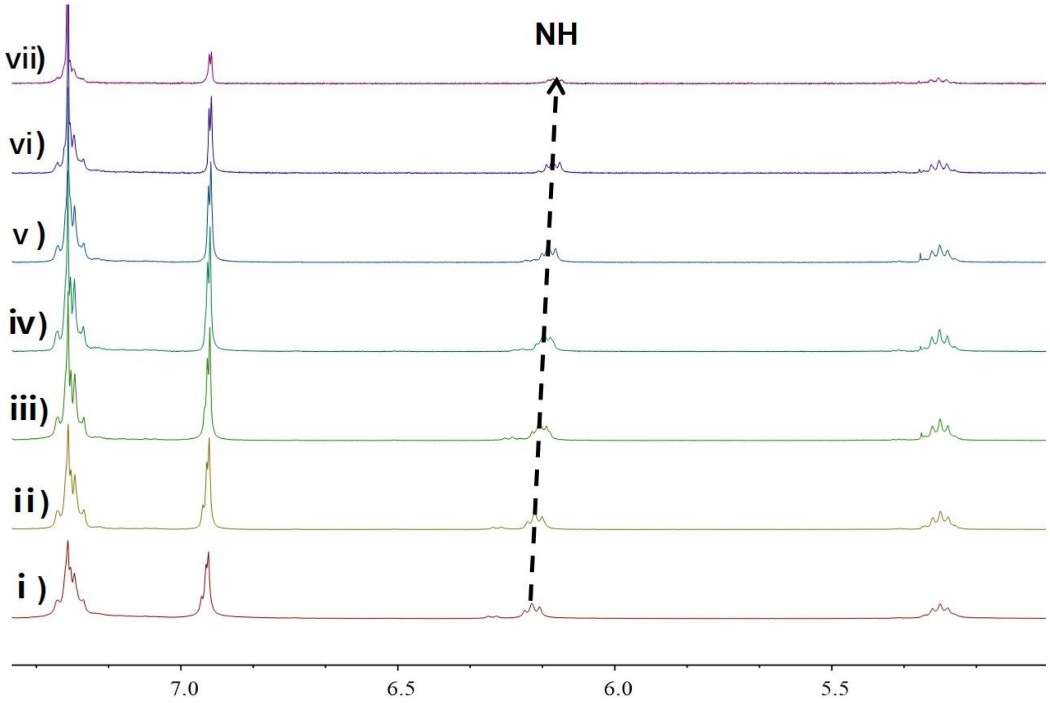

Figure S8. Partial ^{1}H NMR spectra (400 MHz, $CDCl_3$, 298 K) of (*S*)-**2** upon dilution from 30.0 to 2.0 mM. The arrow exhibits the gradual upfield shifting of the NH protons. This finding reveals that intermolecular hydrogen bonds between the amide groups facilitate the supramolecular polymerization process.

*3. Majority-rules for supramolecular polymers derived from (S)-**2**/(R)-**2***

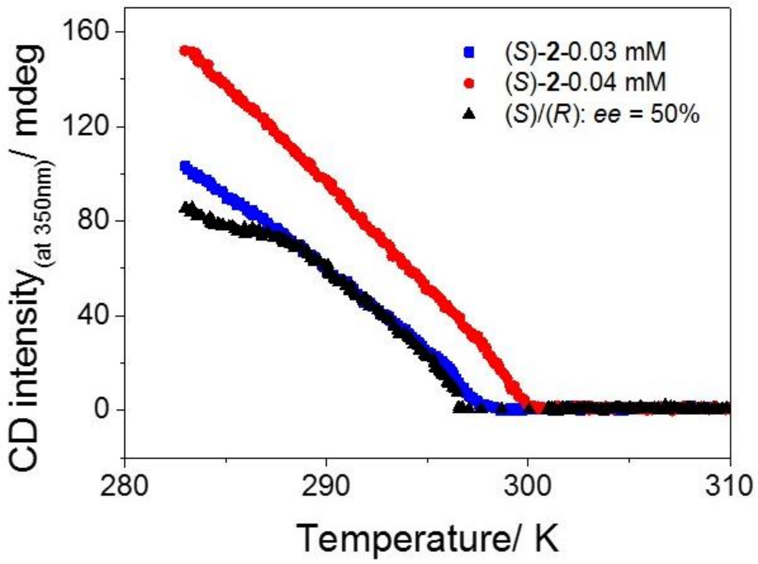

Figure S9. CD cooling curves of (*S*)-**2** at different monomer concentrations [0.03 mM (blue line), and 0.04 mM (red line)], together with the mixture of (*S*)-**2** and (*R*)-**2** (with *ee* = 50 %) at the total concentration of 0.04 mM (black line). For the mixture of (*S*)-**2** and (*R*)-**2** (with *ee* = 50 %), the elongation temperature T_e is determined to be 296.4 K. The value is lower than that of pure (*S*)-**2** at 0.04 mM (T_e = 298.7 K), while similar to that of (*S*)-**2** at 0.03 mM (T_e = 297.6 K). Therefore, it verifies poor mixing capability for the opposite enantiomers, thus giving rise to self-sorted supramolecular homopolymers with low thermal stability.

*4. Supramolecular polymerization behaviors of (S)-**3***

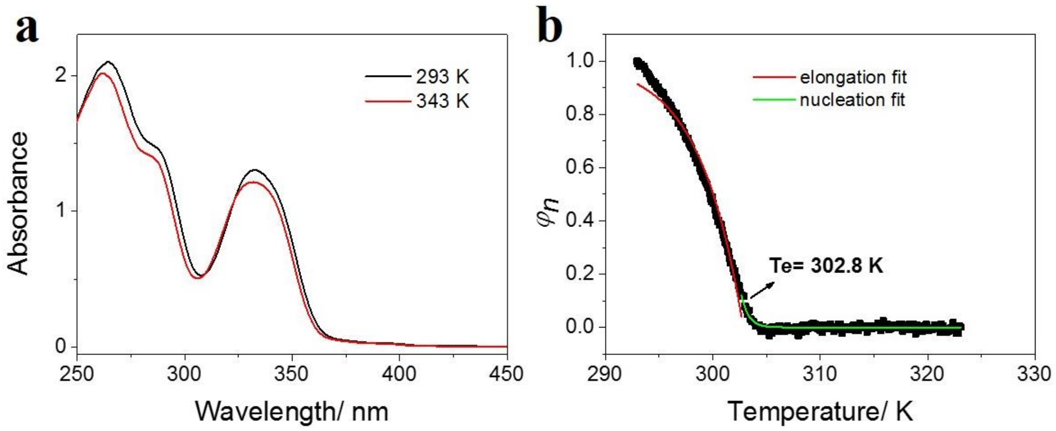

Figure S10. (a) UV–Vis spectra of (*S*)-**3** at 293 K (black line) and 343 K (red line) in MCH/TCE (99 : 1, *v*/*v*, *c* = 0.02 mM). (b) Net helicity φ_n as a function of temperature for (*S*)-**3** in MCH/TCE (99 : 1, *v*/*v*, *c* = 0.02 mM). The red and green lines denote mathematical fitting of the curves by Meijer–Schenning–van der Schoot model.

*5. Synthesis and structural characterization of (S)-**2** and (S)-**3***

Scheme S1. Synthetic Route toward the Platinum(II) Acetylides (*S*)-2 and (*S*)-3

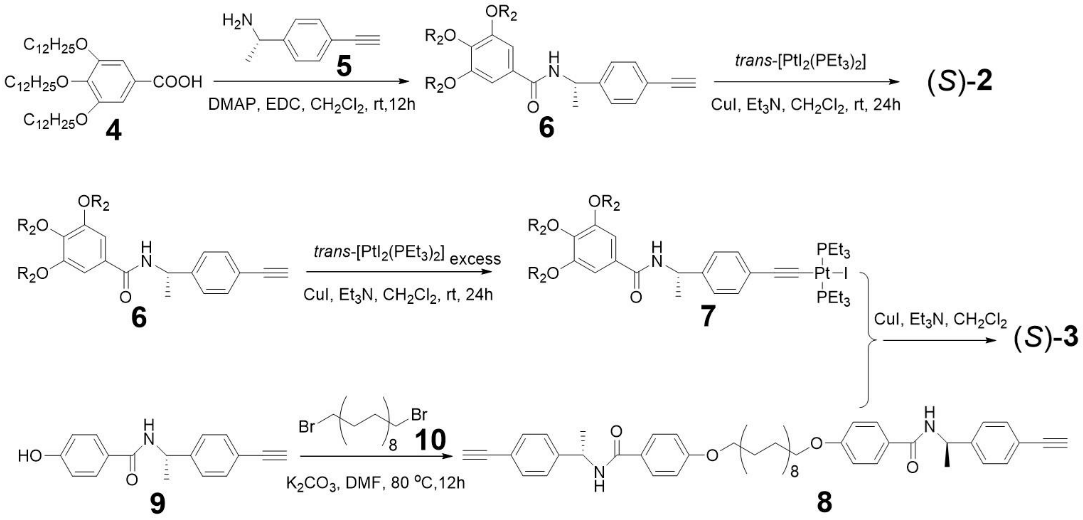

*5.1 Synthesis of **6***

Compounds **4** (2.01 g, 2.98 mmol) and **5** (360 mg, 2.48 mmol), DMAP (510 mg, 4.17 mmol) and EDC·HCl (1.14 g, 5.95 mmol) were mixed in CH_2Cl_2 (50 mL) under nitrogen and stirred at room temperature for 12 h. The solution was extracted with H_2O/CH_2Cl_2 for three times, the solvent was evaporated with a rotary evaporator. The residue was purified by flash column chromatography (silica gel, petroleum ether/CH_2Cl_2, 1 : 3 *v*/*v* as the eluent) to provide **6** as a white solid (1.58 g, 80%). ^{1}H NMR (400 Hz, $CDCl_3$, 298 K): δ 7.48 (d, J = 8.3 Hz, 2H), 7.34 (d, J = 8.2 Hz, 2H), 6.94 (s, 2H), 6.18 (d, J = 7.7 Hz, 1H), 5.40–5.16 (m, 1H), 3.98 (m, 6H), 3.06 (s, 1H), 1.85–1.69 (m, 6H), 1.59 (d, J = 5.6 Hz, 3H), 1.50–1.40 (m, 6H), 1.37–1.21 (m, 39H), 0.88 (t, J = 6.8 Hz, 9H).

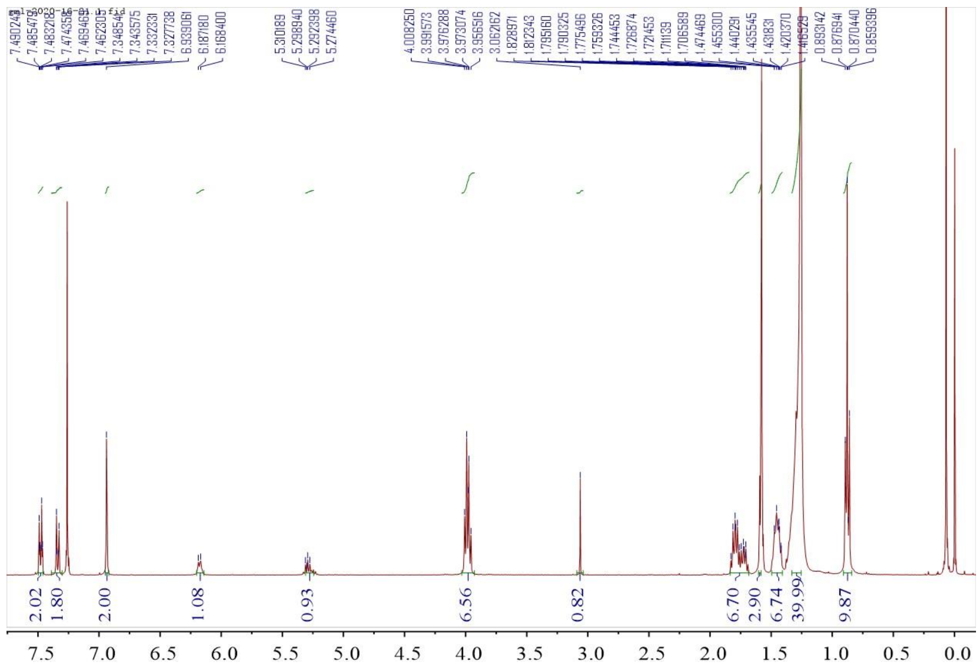

Figure S11. ^{1}H NMR spectrum (400 MHz, $CDCl_3$, 298 K) of **6**.

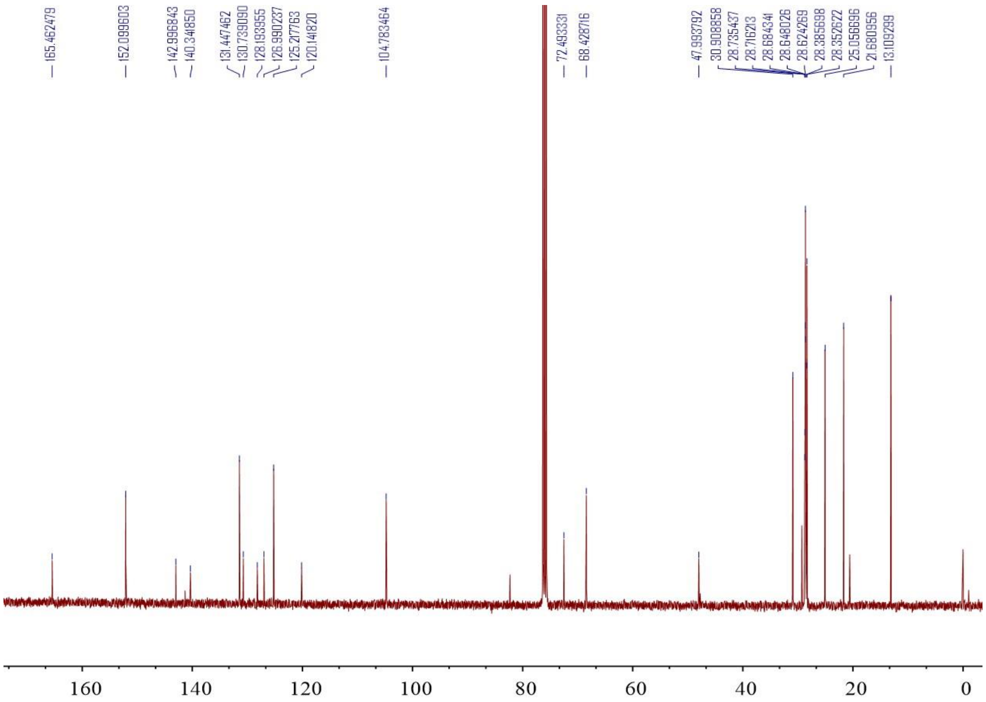

Figure S12. ^{13}C NMR spectrum (101 MHz, $CDCl_3$, 298 K) of **6**.

*5.2. Synthesis of **7***

Trans-$PtI_2(PEt_3)_2$ (1.03 g, 1.50 mmol) and CuI (9.50 mg, 0.10 mmol) were mixed in Et_3N/CH_2Cl_2 (20 mL : 30 mL) under nitrogen atmosphere. Compound **6** (400 mg, 0.50 mmol) in CH_2Cl_2 (10 mL) was added dropwise to the reaction solution. After stirring at room temperature for 24 hours, the solvents were evaporated. The residue was purified by flash column chromatography (silica gel, petroleum ether/CH_2Cl_2, 2 :

3 *v*/*v* as the eluent) to provide **7** as a faint yellow solid (480 mg, 70%). ^{1}H NMR (400 MHz, $CDCl_3$, 298 K): δ 7.26 (m, 4H), 6.94 (s, 2H), 6.14 (d, *J* = 7.7 Hz, 1H), 5.25 (m, 1H), 4.07 – 3.86 (m, 6H), 2.21 (m, 12H), 1.77 (m, 6H), 1.58 (d, *J* = 6.9 Hz, 3H), 1.45 (m, 6H), 1.26 (m, 48H), 1.21 – 1.11 (m, 18H), 0.88 (t, *J* = 6.8 Hz, 9H).

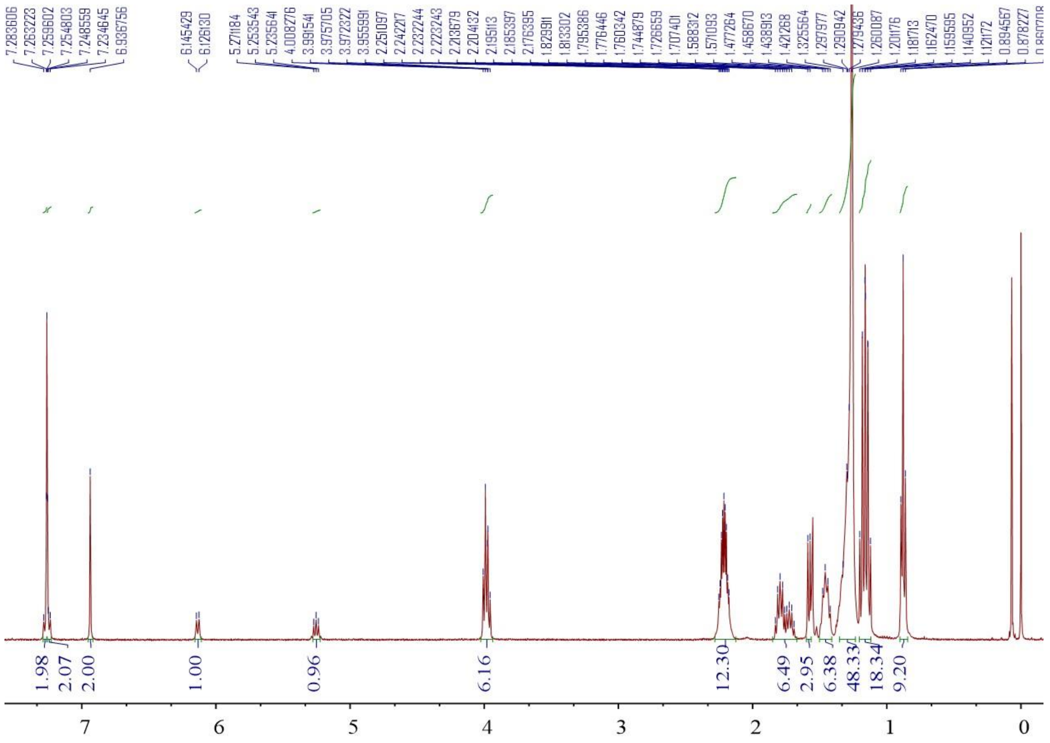

Figure S13. ^{1}H NMR spectrum (400 MHz, $CDCl_3$, 298 K) of **7**.

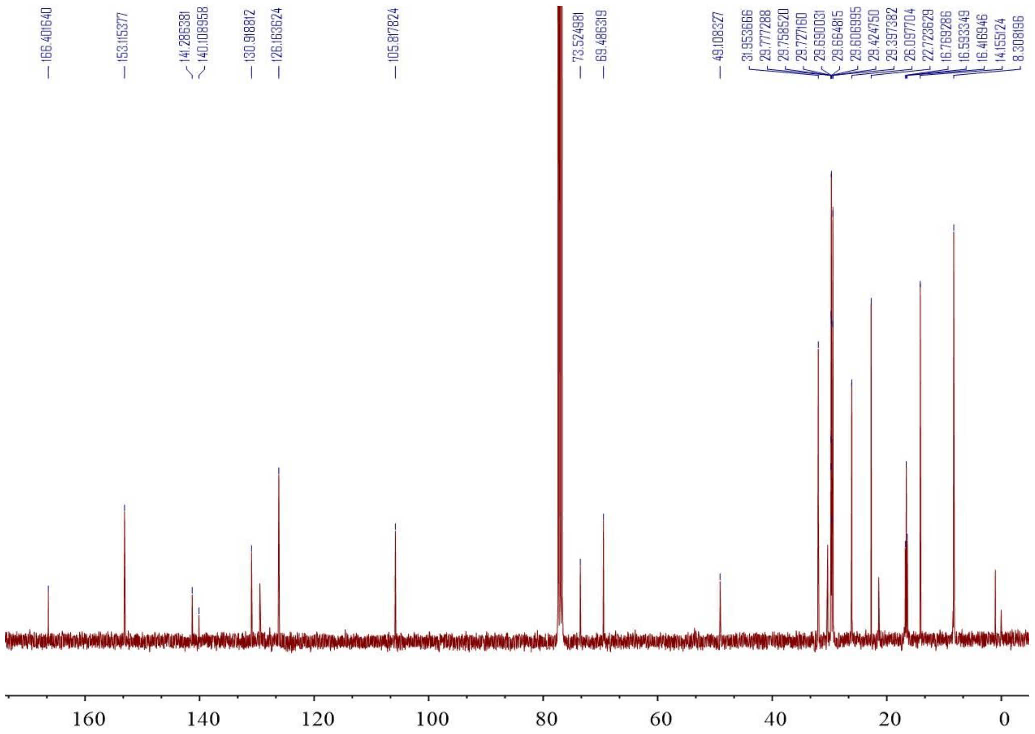

Figure S14. ^{13}C NMR spectrum (101 MHz, $CDCl_3$, 298 K) of **7**.

5.3. Synthesis of ***8***

Compounds **9** (320 mg, 1.21 mmol) and K_2CO_3 (556 mg, 4.03 mmol) were mixed in DMF (50 mL) under nitrogen and stirred at 353 K. Compound **10** (237 mg, 0.58 mmol) dissolved in DMF was added to the reaction solution. After stirring at 353 K for 12h, the solvent DMF was evaporated. The solution was extracted with H_2O/CH_2Cl_2 for three times, the solvent was evaporated with a rotary evaporator. The residue was purified by flash column chromatography (silica gel, CH_3OH/CH_2Cl_2, 0.5 : 99.5 *v*/*v* as the eluent) to provide **8** as a white solid (370 mg, 82%). ^{1}H NMR (400 MHz, $CDCl_3$, 298 K): δ 7.71 (dd, J = 8.9, 2.6 Hz, 4H), 7.47 (dd, J = 8.4, 3.1 Hz, 4H), 7.34 (d, J = 7.8 Hz, 4H), 6.88 (d, J = 8.8 Hz, 4H), 6.22 (d, J = 7.7 Hz, 2H), 5.38 – 5.20 (m, 2H), 3.98 (t, J = 6.6 Hz, 4H), 3.05 (m, 2H), 1.78 (m, 4H), 1.58 (d, J = 4.5 Hz, 6H), 1.44 (m, 4H), 1.36 – 1.23 (m, 24H).

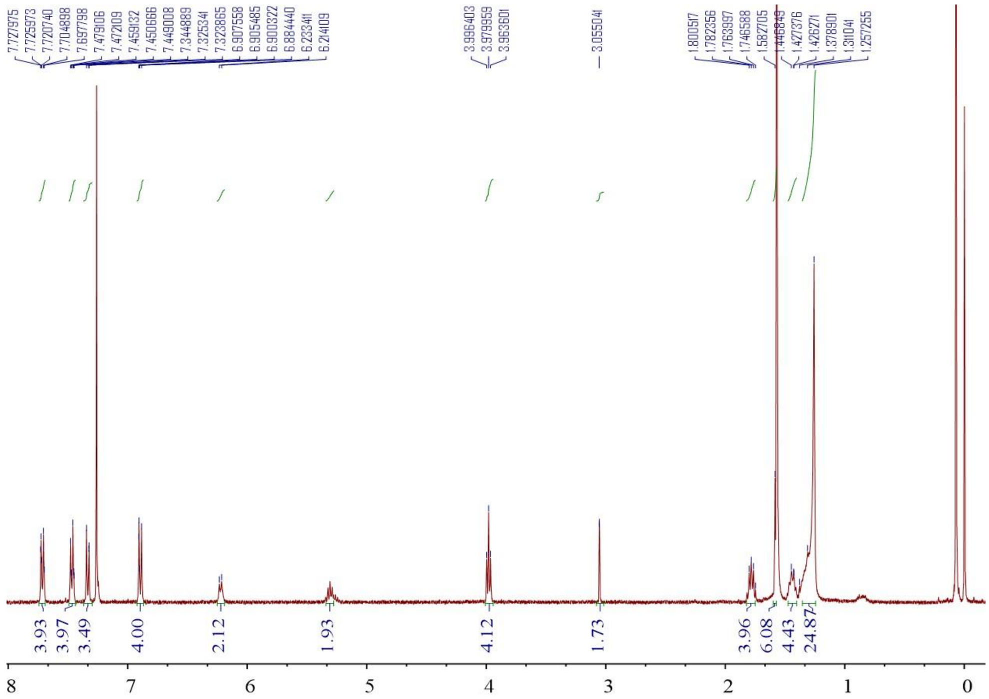

Figure S15. ^{1}H NMR spectrum (400 MHz, $CDCl_3$, 298 K) of **8**.

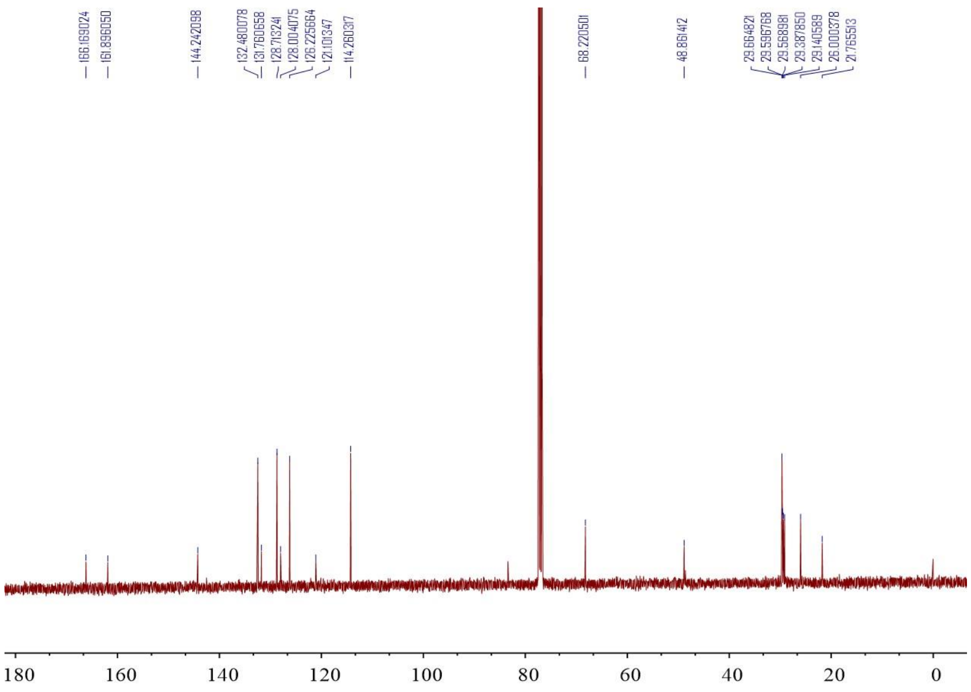

Figure S16. ^{13}C NMR spectrum (101 MHz, $CDCl_3$, 298 K) of **8**.

*5.4. Structural characterization of (S)-**2***

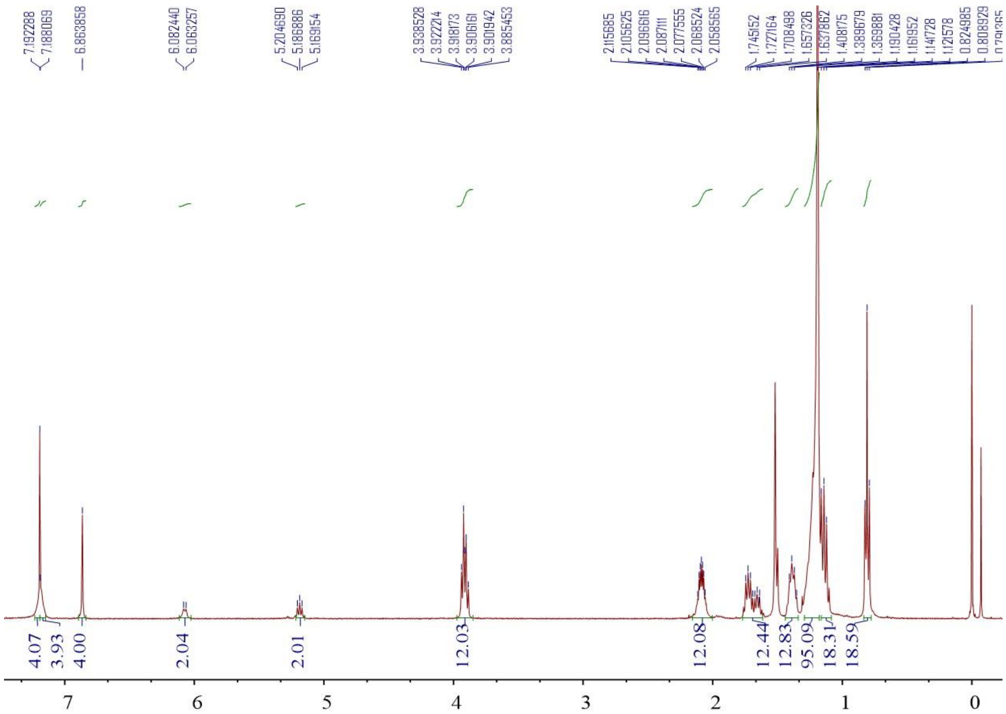

Figure S17. ^{1}H NMR spectrum (400 MHz, $CDCl_3$, 298 K) of (*S*)-**2**.

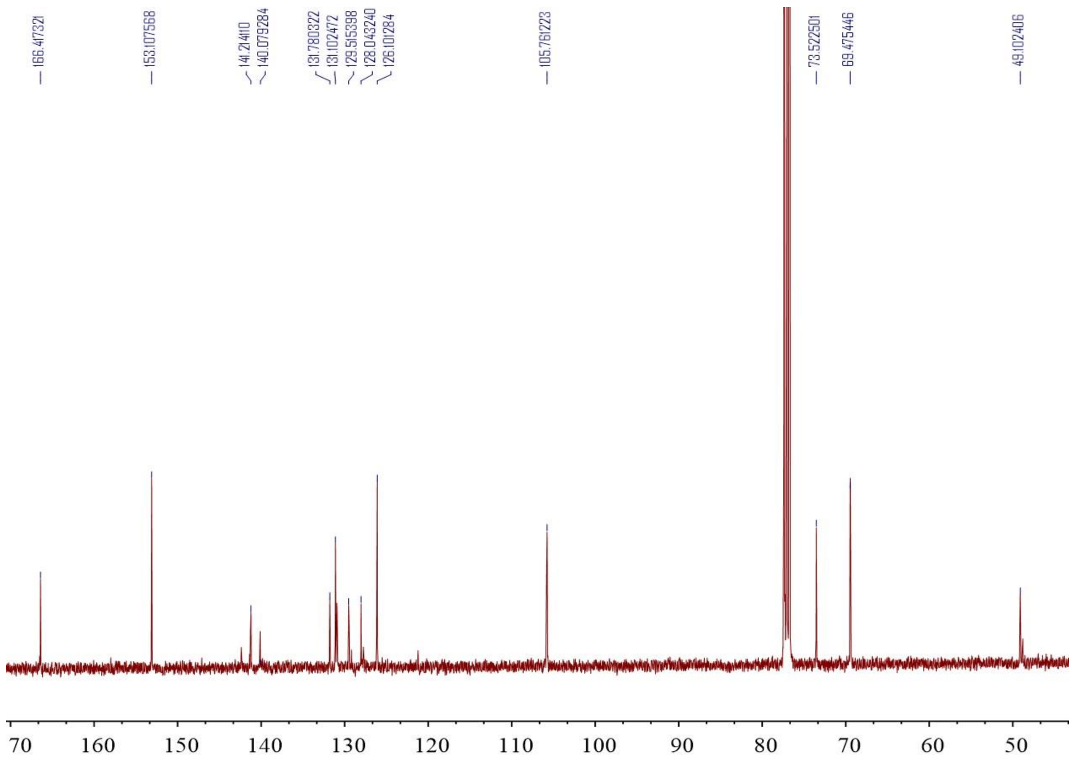

Figure S18. ^{13}C NMR spectrum (101 MHz, $CDCl_3$, 298 K) of (*S*)-**2**.

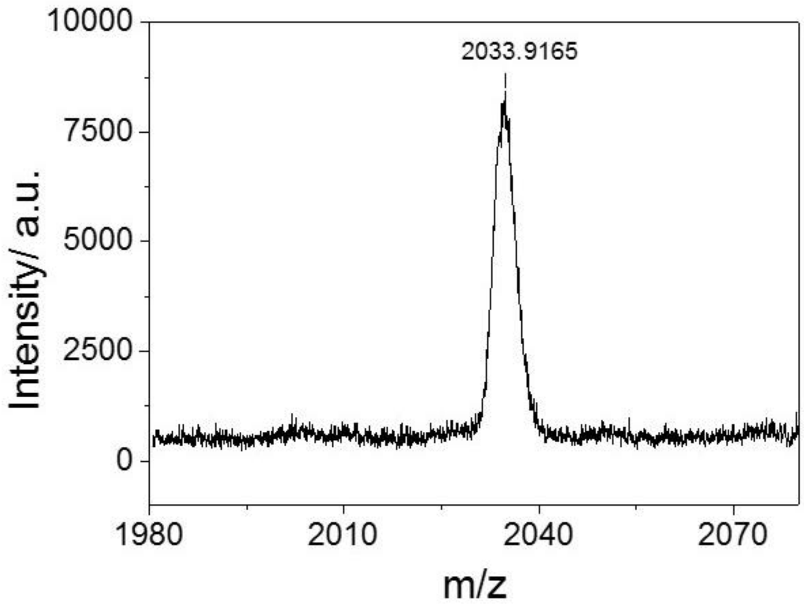

Figure S19. MALDI-TOF mass spectrum of (*S*)-**2**.

*5.5. Structural characterization of (S)-**3***

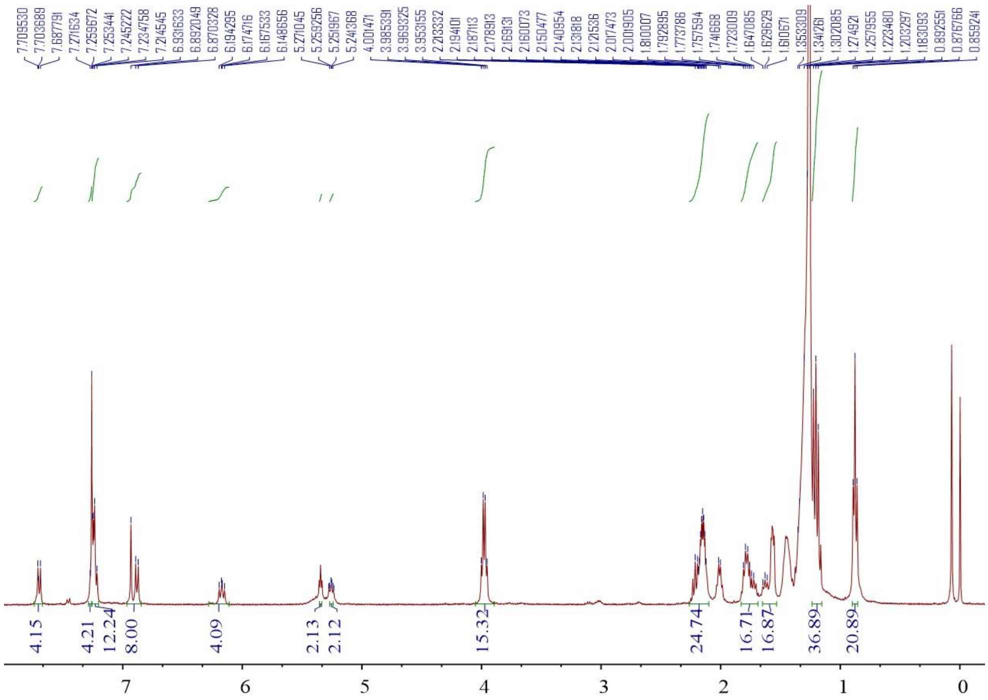

Figure S20. ^{1}H NMR spectrum (400 MHz, $CDCl_3$, 298 K) of (*S*)-**3**.

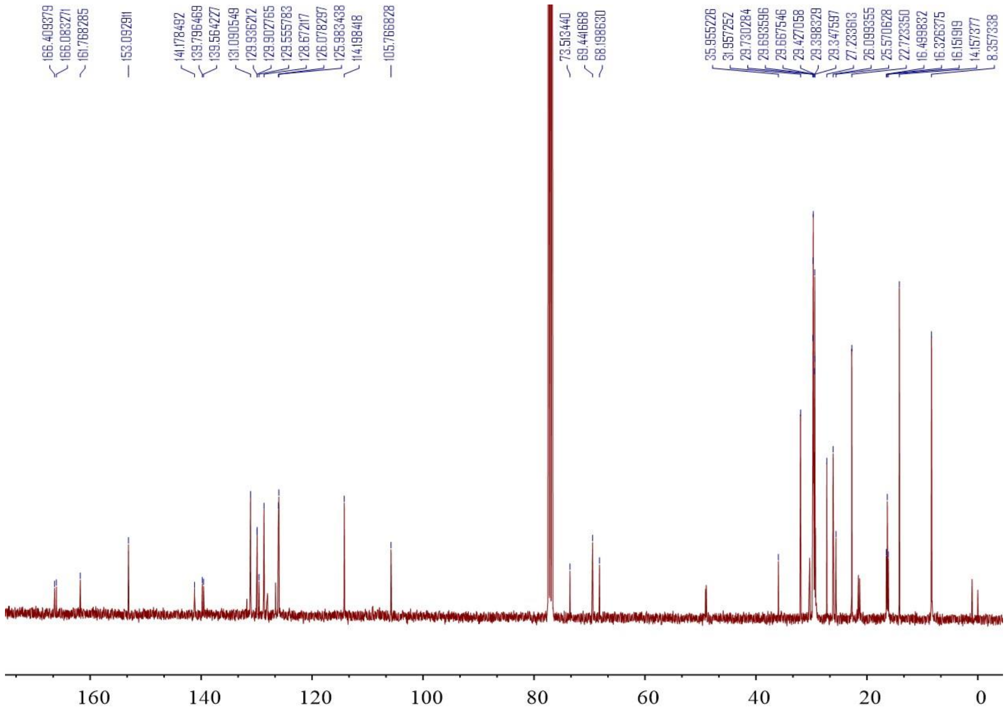

Figure S21. ^{13}C NMR spectrum (101 MHz, $CDCl_3$, 298 K) of (*S*)-**3**.

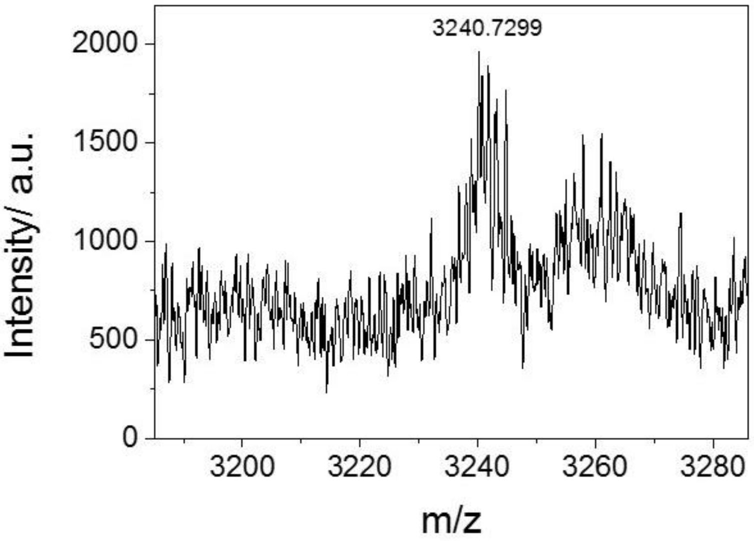

Figure S22. MALDI-TOF mass spectrum of (*S*)-**3**.
